# Supplementary material for: Effects of myosin variants on interacting-heads motif explain distinct hypertrophic and dilated cardiomyopathy phenotypes
Source: eLife. 2017 Jun 13;6:e24634. doi: 10.7554/eLife.24634 (PMC5469618; doi:10.7554/eLife.24634)
Supplement: Supplementary file 1. — DOI: http://dx.doi.org/10.7554/eLife.24634.029 [file elife-24634-supp1.docx]

**Supplementary file 1.** **Domains of human β-cardiac myosin**

| Domain^a^ | Residues  in human β-cardiac muscle sequence  P12883 | Homologous residues in chicken skeletal muscle sequence P13538 | Homologous residues  in tarantula skeletal muscle sequence  KT619079 |
| --- | --- | --- | --- |
| S1 |  |  |  |
| SH3 | **M1-Q79** | M1-M81 | M1-V74 |
| Nucleotide binding pocket |  |  |  |
| ATP-binding I | **N126-Y134** | N128-Y136 | N121-Y129 |
| ATP-binding II (P-loop) | **G178-N187** | G180-N189 | G173-N182 |
| ATP-binding III (Switch 1) | **N238-G245** | N241-G248 | N235-G242 |
| ATP-binding IV | **E269** | E272 | E266 |
| ATP-binding V (Switch 2) | **D461- N471** | D464- N474 | D458- N468 |
| Actin-myosin interface |  |  |  |
| 25/50 junction (Loop 1) | **D203-G214** | E205-G217 | P198-A211 |
| Actin-interface I (CM-Loop) | **H401-N416** | Y404-T419 | K398-N413 |
| Actin-interface II (H-Loop) | **P527-H556** | P530-H559 | P524-H553 |
| Actin-interface III (Loop 3) | **Q564-F577** | Q567-F580 | V561-F575 |
| Helix-loop-helix | **W593-Q610** | W596-Q613 | W591-K608 |
| Strut | **D599-E603** | D602-E606 | D597-D601 |
| 50/20 junction (Loop 2) | **A622-Q645** | A625-Q648 | E620-Q647 |
| C-Loop (Loop 4) | **N361-E380** | N364-V383 | E358-E377 |
| I-Loop | **L301-A326** | L304-Q329 | L298-A323 |
| Relay | **N490-D516** | N493-D519 | N487-D513 |
| SH2 Helix | **D685-C695** | E688-C698 | D687-C697 |
| SH1 Helix | **V698-F709** | V701-F712 | V700-F711 |
| Converter | **P710-R777** | P713-R780 | P712-R778 |
| Pliant | **D778-R783** | D781-E786 | D779-K784 |
| Regulatory Domain |  |  |  |
| Neck (Lever arm) | **I784-K837** | I787-K840 | I785-K838 |
| ELC binding interface | **L781-L804** | L784-M807 | L782-L805 |
| RLC binding interface | **G823-E846** | N826-E849 | T824-E847 |
| Rod |  |  |  |
| S2 | **P838-S1261** | P841-T1264 | P839-G1262 |
| Ring 1 | **E894-D906** | E897-D909 | E895-T907 |
| Ring 2 | **E921-E935** | E924-E938 | E922-N936 |
| Ring 3 | **E944-E958** | E947-E961 | D945-Q959 |
| LMM | **K1262-E1935** | K1265-E1939 | K1263-E1953 |
| Skip 1 | **T1188** | T1191 | T1189 |
| Skip 2 | **E1385** | E1388 | 1386 |
| Skip 3 | **E1582** | E1585 | 1583 |
| Skip 4 | **G1807** | G1810 | G1808 |

^a^Locations and interaction types follow the nomenclature of (Alamo et al., 2016).
