# Supplementary material for: Effects of myosin variants on interacting-heads motif explain distinct hypertrophic and dilated cardiomyopathy phenotypes
Source: eLife. 2017 Jun 13;6:e24634. doi: 10.7554/eLife.24634 (PMC5469618; doi:10.7554/eLife.24634)
Supplement: Supplementary file 2. — DOI: http://dx.doi.org/10.7554/eLife.24634.030 [file elife-24634-supp2.docx]

**Supplementary file 2. Intra- and inter-molecular interactions sequences involved in human β-cardiac myosin interacting-heads motif (IHM) PDB 5TBY**

| **Interaction type** | **Interaction^a^** | **S2** | **Blocked head** | | **Free head** | |
| --- | --- | --- | --- | --- | --- | --- |
|  |  |  | **Myosin heavy chain**  **(MYH7)** | **Light chains (MYL2/MYL3)** | **Myosin heavy chain**  **(MYL7)** | **Light chains (MYL2/MYL3)** |
| **IHM “stabilizing”** | **“a”** | **S2 Ring 2**  915-931 |  |  | **Loop 2**  622-646 |  |
|  | **“d.1”** |  | **CM-loop**  401-416 |  | **Near P-Loop**  168-171  **Near Switch 2**  442**-**454 |  |
|  | **“d.2”** |  | **Near I-Loop**  295-305 |  | **Converter**  732-741 |  |
|  |  |  | **C-Loop (Loop 4)**  365-383 |  | **Converter**  716-723,  759-766  **Relay**  483-515 |  |
|  | **“e”** |  | **Near Loop 2**  606-624  **Near C-loop**  386-394 |  |  | **ELC**  138-155 |
| **IHM “priming”** | **“f.1”** | **S2 Ring 1 (BH)**  889-913 | **Loop 2**  622-646 |  |  |  |
|  | **“f.2”** | **S2 Ring 1(FH)**  889-913 | **H-Loop**  524-538  **Near Loop 2**  651-663 |  |  |  |
|  | **“g”** | **S2 (FH)**  858-884 | **Near Switch 2**  447-454 |  |  |  |
|  |  |  |  | **ELC**  175-180 |  |  |
| **IHM “anchoring”** | **“h”** | **Adjacent myosin S2^e^?** | **SH3**  1-79 |  |  |  |
|  | **“i”** |  | **Converter^b^**  716-723 | **ELC**  121-155 |  |  |
|  |  | **Adjacent myosin S2^e^**  **?** |  | **ELC**  121-155  180-190 |  |  |
|  | **“j”** | **Adjacent myosin S2^e^**  **?** | **Relay**  483-516 |  |  |  |
|  |  |  | **Converter**  728**-**762 | **ELC^c^**  138-142 |  |  |
| **IHM**  **“scaffolding”** | **ELC-MHC** |  |  |  | **MHC-ELC interface^d^**  777-808 | **ELC**  59-64,81-91,125-140, 156-172, 188-195 |
|  | **ELC-MHC** |  | **MHC-ELC interface^d^**  716-762 | **ELC**  121-155 |  |  |
|  |  |  | 774-799 | 81-89, 157-173,192-195 |  |  |
|  |  |  | 804-815 | 59-69 |  |  |
| **IHM**  **“scaffolding”** | **RLC-MHC** |  |  |  | **MHC-RLC interface**  800-846 | **RLC**  22-35,52-72,94-109, 122-138, 155-166 |
|  | **RLC-MHC** |  |  | **RLC**  21-25 | **MHC-RLC interface**  841-845 |  |
|  | **RLC-MHC** |  | **MHC-RLC interface**  826-841 |  |  | **RLC**  8-23 |
|  | **RLC-MHC** |  | **MHC-RLC interface**  803-846 | **RLC**  19-31,56-60,94-108, 127-141,154-166 |  |  |
| **IHM “regulating”** | **RLC-RLC** |  |  | **RLC**  22-41,54-61,164-166 |  | **RLC**  7-31,79-88 |

^a^IHM interactions follows the nomenclature of (Alamo et al., 2016). ^b^Interaction "i" is established between BH-ELC and the S2 of a neighbor IHM at unknown sequences (designated as “?”). The PISA analysis for interaction “i” identifies that three variants (716, 719, 723) on the BH-MHC converter that also make a contact with the ELC loop. ^c^Interaction "j" established between BH-converter and the S2 of a neighbor IHM at unknown sequences (designated as “?”). The PISA analysis for this interaction identifies two variants (736, 741) on the BH-MHC converter that also make a contact with the ELC loop. ^d^Converter + pliant + ELC binding interface. **^e^** This refers to the S2 on a neighbor myosin molecule, following the nomenclature of (Alamo et al., 2016). IHM interactions are colored as previously: priming = green, anchoring = orange, stabilizing = magenta, scaffolding = white.
