# Supplementary material for: Effects of myosin variants on interacting-heads motif explain distinct hypertrophic and dilated cardiomyopathy phenotypes
Source: eLife. 2017 Jun 13;6:e24634. doi: 10.7554/eLife.24634 (PMC5469618; doi:10.7554/eLife.24634)
Supplement: Supplementary file 3. — DOI: http://dx.doi.org/10.7554/eLife.24634.031 [file elife-24634-supp3.docx]

**Supplementary File 3. HCM variants cluster on residues involved in IHM-related inter- and intra-molecular interactions.**

***Table 3a - Pathogenic variants.*** For each of the four main IHM interactions (IHM priming, anchoring, stabilizing and scaffolding), the number of distinct pathogenic variants (Table 1, in the main text) affecting interacting residues (of a total of 40 variants) is shown. The length of the interaction site (no of amino acid residues; total protein length = 1935) is used to determine the proportion of variants that would be expected to lie in the region of interest under the null (a uniform distribution), and the rates are compared with a binomial test.

| interaction | variants in interaction site | rate | length of interaction site (aa) | expected rate | rate ratio | p_binom_ |
| --- | --- | --- | --- | --- | --- | --- |
| **all IHM interactions** | 31 | 0.775 | 447 | 0.2310 | 3.35 | 5.25e-13 |
| priming | 5 | 0.125 | 113 | 0.0584 | 2.14 | 8.19e-02 |
| anchoring | 12 | 0.300 | 156 | 0.0806 | 3.72 | 4.91e-05 |
| stabilizing | 24 | 0.600 | 189 | 0.0977 | 6.14 | 7.41e-15 |
| scaffolding | 13 | 0.325 | 120 | 0.0620 | 5.24 | 4.90e-07 |
| **MD functional residues** | 16 | 0.400 | 194 | 0.1000 | 4.00 | 6.14e-07 |

***Table 3b - Likely pathogenic variants.*** Equivalent data shown for 95 likely pathogenic variants (Table 2, in the main text). The clustering in the IHM priming and stabilizing regions is replicated.

| interaction | variants in interaction site | rate | length of interaction site (aa) | expected rate | rate ratio | p_binom_ |
| --- | --- | --- | --- | --- | --- | --- |
| **all IHM interactions** | 42 | 0.442 | 447 | 0.2310 | 1.91 | 7.04e-06 |
| priming | 12 | 0.126 | 113 | 0.0584 | 2.16 | 1.27e-02 |
| anchoring | 12 | 0.126 | 156 | 0.0806 | 1.56 | 1.27e-01 |
| stabilizing | 24 | 0.253 | 189 | 0.0977 | 2.59 | 1.08e-05 |
| scaffolding | 11 | 0.116 | 120 | 0.0620 | 1.87 | 5.00e-02 |
| **MD functional residues** | 23 | 0.242 | 194 | 0.1000 | 2.42 | 5.11e-05 |

***Table 3c - All pathogenic / likely pathogenic variants.*** Shows equivalent statistics when 135 pathogenic and likely pathogenic variants, are analyzed in combination. This table appears in the main text as Table 4, duplicated here for convenience.

| interaction | variants in interaction site | rate | length of interaction site (aa) | expected rate | rate ratio | p_binom_ |
| --- | --- | --- | --- | --- | --- | --- |
| **all IHM interactions** | 73 | 0.541 | 447 | 0.2310 | 2.34 | 7.95e-15 |
| priming | 17 | 0.126 | 113 | 0.0584 | 2.16 | 2.64e-03 |
| anchoring | 24 | 0.178 | 156 | 0.0806 | 2.21 | 2.11e-04 |
| stabilizing | 48 | 0.356 | 189 | 0.0977 | 3.64 | 5.37e-16 |
| scaffolding | 24 | 0.178 | 120 | 0.0620 | 2.87 | 2.97e-06 |
| **MD functional residues** | 39 | 0.289 | 194 | 0.1000 | 2.89 | 7.87e-10 |

***Table 3d - All interactions.*** Pathogenic and likely pathogenic variants are combined (total = 135), to empower interrogation of each individual interaction motif. Interactions refer to definitions given in Supplementary File 2, and full details of all variants are shown in Tables 1, 2.

| interaction | variants in interaction site | rate | length of interaction site (aa) | expected rate | rate ratio | p_binom_ |
| --- | --- | --- | --- | --- | --- | --- |
| i (bh) | 5 | 0.03700 | 8 | 0.00413 | 8.960 | 2.68e-04 |
| d1 (fh) | 9 | 0.06670 | 17 | 0.00879 | 7.590 | 3.60e-06 |
| g (bh) | 4 | 0.02960 | 8 | 0.00413 | 7.170 | 2.52e-03 |
| d1 (bh) | 7 | 0.05190 | 16 | 0.00827 | 6.280 | 1.45e-04 |
| d2 (fh) | 22 | 0.16300 | 59 | 0.03050 | 5.340 | 1.72e-10 |
| a (tail) | 6 | 0.04440 | 17 | 0.00879 | 5.050 | 1.32e-03 |
| j (bh) | 18 | 0.13300 | 69 | 0.03570 | 3.730 | 1.69e-06 |
| elc-mhc (bh) | 22 | 0.16300 | 85 | 0.04390 | 3.710 | 1.19e-07 |
| f1 (tail) | 6 | 0.04440 | 25 | 0.01290 | 3.440 | 8.50e-03 |
| f2 (tail) | 6 | 0.04440 | 25 | 0.01290 | 3.440 | 8.50e-03 |
| f2 (bh) | 5 | 0.03700 | 28 | 0.01450 | 2.550 | 4.73e-02 |
| elc-mhc (fh) | 5 | 0.03700 | 32 | 0.01650 | 2.240 | 7.44e-02 |
| g (tail) | 2 | 0.01480 | 27 | 0.01400 | 1.060 | 7.13e-01 |
| e (bh) | 2 | 0.01480 | 28 | 0.01450 | 1.020 | 7.23e-01 |
| rlc-mhc (bh) | 3 | 0.02220 | 44 | 0.02270 | 0.978 | 1.00e+00 |
| d2 (bh) | 2 | 0.01480 | 30 | 0.01550 | 0.955 | 1.00e+00 |
| rlc-mhc (fh) | 3 | 0.02220 | 47 | 0.02430 | 0.914 | 1.00e+00 |
| h (bh) | 1 | 0.00741 | 79 | 0.04080 | 0.182 | 4.72e-02 |
| f1 (bh) | 0 | 0.00000 | 25 | 0.01290 | 0.000 | 4.27e-01 |
| a (fh) | 0 | 0.00000 | 25 | 0.01290 | 0.000 | 4.27e-01 |

***Table 3e - All MD functional sites.*** Pathogenic and likely pathogenic variants are combined as above (total = 135), to empower interrogation of individual motor domain functional sites. Full variant details are shown in Tables 1, 2.

| interaction | variants in interaction site | rate | length of interaction site (aa) | expected rate | rate ratio | p_binom_ |
| --- | --- | --- | --- | --- | --- | --- |
| converter | 21 | 0.1560 | 68 | 0.0351 | 4.44 | 1.25e-08 |
| relay | 6 | 0.0444 | 27 | 0.0140 | 3.17 | 1.21e-02 |
| actin-myosin | 9 | 0.0667 | 60 | 0.0310 | 2.15 | 3.96e-02 |
| nucleotideBinding | 3 | 0.0222 | 39 | 0.0202 | 1.10 | 7.55e-01 |

***Table 3f - Tabulating differential impact of variants located on the free head (FH) and blocked head (BH).*** Pathogenic and likely pathogenic variants are analyzed in combination. Full variant details are shown in Tables 1, 2.

| interaction | variants in interaction site | rate | length of interaction site (aa) | expected rate | rate ratio | p_binom_ |
| --- | --- | --- | --- | --- | --- | --- |
| bh | 52 | 0.385 | 362 | 0.1870 | 2.06 | 9.32e-08 |
| fh | 39 | 0.289 | 171 | 0.0884 | 3.27 | 1.93e-11 |
| tail | 14 | 0.104 | 69 | 0.0357 | 2.91 | 3.57e-04 |
