# Supplementary material for: Effects of myosin variants on interacting-heads motif explain distinct hypertrophic and dilated cardiomyopathy phenotypes
Source: eLife. 2017 Jun 13;6:e24634. doi: 10.7554/eLife.24634 (PMC5469618; doi:10.7554/eLife.24634)
Supplement: Supplementary file 4. — DOI: http://dx.doi.org/10.7554/eLife.24634.032 [file elife-24634-supp4.docx]

**Supplementary file 4. DCM-causing variants cluster in distinct regions of *MYH7* from HCM-causing variants.**

***Table 4a - Assessing the distribution of DCM-causing variants across IHM and MD functional residues.*** For each of the main IHM interactions (IHM forming, anchoring, stabilizing, and scaffolding) and the motor domain functional sites, the number of distinct pathogenic or likely pathogenic DCM variants (Table 3, in the main text) affecting interacting or functional residues (of a total of 27 variants) is shown. The length of the interaction site (number of amino acid residues; total protein length = 1935) is used to determine the proportion of variants that would be expected to lie in the region of interest under the null (a uniform distribution), and the rates are compared with a binomial test. This table appears in the main text as Table 5, duplicated here for convenience.

| interaction | variants in interaction site | rate | length of interaction site (aa) | expected rate | rate ratio | p_binom_ |
| --- | --- | --- | --- | --- | --- | --- |
| **all IHM interactions** | 7 | 0.259 | 447 | 0.2310 | 1.120 | 0.6550 |
| priming | 5 | 0.185 | 113 | 0.0584 | 3.170 | 0.0186 |
| anchoring | 0 | 0.000 | 156 | 0.0806 | 0.000 | 0.1650 |
| stabilizing | 1 | 0.037 | 189 | 0.0977 | 0.379 | 0.5120 |
| scaffolding | 1 | 0.037 | 120 | 0.0620 | 0.597 | 1.0000 |
| **MD functional residues** | 7 | 0.259 | 210 | 0.1090 | 2.380 | 0.0222 |

***Table 4b - Sites of IHM-priming interactions are over-represented amongst DCM-causing variants.*** The distribution of DCM-causing variants is assessed across individual reaction motifs. Only the "f" interactions, that are found on the blocked head and tail and contribute to IHM priming, achieve statistical significance. Full variant details are shown in Table 3.

| interaction | variants in interaction site | rate | length of interaction site (aa) | expected rate | rate ratio | p_binom_ |
| --- | --- | --- | --- | --- | --- | --- |
| f2 | 5 | 0.1850 | 53 | 0.02740 | 6.750 | 0.000752 |
| f1 | 2 | 0.0741 | 50 | 0.02580 | 2.870 | 0.154000 |
| d2 | 1 | 0.0370 | 89 | 0.04600 | 0.804 | 1.000000 |
| elc-mhc | 1 | 0.0370 | 89 | 0.04600 | 0.804 | 1.000000 |
| g | 0 | 0.0000 | 35 | 0.01810 | 0.000 | 1.000000 |
| h | 0 | 0.0000 | 79 | 0.04080 | 0.000 | 0.627000 |
| i | 0 | 0.0000 | 8 | 0.00413 | 0.000 | 1.000000 |
| j | 0 | 0.0000 | 69 | 0.03570 | 0.000 | 1.000000 |
| a | 0 | 0.0000 | 42 | 0.02170 | 0.000 | 1.000000 |
| d1 | 0 | 0.0000 | 33 | 0.01710 | 0.000 | 1.000000 |
| e | 0 | 0.0000 | 28 | 0.01450 | 0.000 | 1.000000 |
| rlc-mhc | 0 | 0.0000 | 47 | 0.02430 | 0.000 | 1.000000 |

***Table 4c - DCM-causing variants cluster are enriched at the nucleotide binding pocket.*** The distribution of DCM-causing variants is assessed for individual motor domain functional regions. Full variant details are shown in Table 3.

| interaction | variants in interaction site | rate | length of interaction site (aa) | expected rate | rate ratio | p_binom_ |
| --- | --- | --- | --- | --- | --- | --- |
| nucleotideBinding | 5 | 0.1850 | 39 | 0.0202 | 9.16 | 0.000185 |
| actin-myosin | 2 | 0.0741 | 60 | 0.0310 | 2.39 | 0.204000 |
| relay | 0 | 0.0000 | 27 | 0.0140 | 0.00 | 1.000000 |
| converter | 0 | 0.0000 | 68 | 0.0351 | 0.00 | 1.000000 |
|  |  |  |  |  |  |  |

***Table 4d - DCM-causing variants grouped by myosin head state.*** The number of DCM-causing variants that impact IHM residues differs for paired myosins depending whether the head is free or blocked in the pair. Here the number of DCM-causing variants is tabulated for IHM residues found on the free head (FH), blocked head (BH), and myosin tail. Full variant details are shown in Table 3.

| interaction | variants in interaction site | rate | length of interaction site (aa) | expected rate | rate ratio | p_binom_ |
| --- | --- | --- | --- | --- | --- | --- |
| tail | 2 | 0.0741 | 69 | 0.0357 | 2.080 | 0.25 |
| bh | 5 | 0.1850 | 362 | 0.1870 | 0.989 | 1.00 |
| fh | 1 | 0.0370 | 171 | 0.0884 | 0.419 | 0.51 |
