# Supplementary material for: Effects of myosin variants on interacting-heads motif explain distinct hypertrophic and dilated cardiomyopathy phenotypes
Source: eLife. 2017 Jun 13;6:e24634. doi: 10.7554/eLife.24634 (PMC5469618; doi:10.7554/eLife.24634)
Supplement: Supplementary file 5. — DOI: http://dx.doi.org/10.7554/eLife.24634.033 [file elife-24634-supp5.docx]

**Supplementary file 5. Variants Clustered on the Myosin Mesa**

***Table 5 - Assessing the enrichment of HCM and DCM-causing variants on the mesa.*** The mesa is defined as the 277 residue surface observed in the pre-stroke state, as reported in (Homburger et al., 2016), which represents 0.14% of the MYH7 protein. The proportion of HCM & DCM causing variants that fall on the mesa surface is shown, and compared with the expected proportion using the binomial test. Full variant details are shown in Tables 1-3 (in the main text).

|  | n variants on mesa | proportion of variants on mesa | enrichment | p_binom_ |
| --- | --- | --- | --- | --- |
| HCM, pathogenic | 18 | 0.450 | 3.150 | 2.98e-06 |
| HCM, likely pathogenic | 34 | 0.358 | 2.500 | 1.55e-07 |
| HCM, all pathogenic | 52 | 0.385 | 2.690 | 3.89e-12 |
| DCM, all pathogenic | 3 | 0.111 | 0.776 | 7.89e-01 |
