# Supplementary material for: Effects of myosin variants on interacting-heads motif explain distinct hypertrophic and dilated cardiomyopathy phenotypes
Source: eLife. 2017 Jun 13;6:e24634. doi: 10.7554/eLife.24634 (PMC5469618; doi:10.7554/eLife.24634)
Supplement: Supplementary file 6. — DOI: http://dx.doi.org/10.7554/eLife.24634.034 [file elife-24634-supp6.docx]

**Supplementary file 6. Comparison of prevalence of rare (ExAC global AF < 1x10^-4^) missense variants in *MYH7* in 6112 HCM cases and ExAC controls** (median number genotype per site = 33364). Prevalences are compared using the binomial test. In the absence of individual-level genotypes for ExAC, the odds ratio is computed under the assumption that no individual contains more than one variant, so the number of variant carriers / non-carriers is extrapolated from the variant prevalence. The etiological fraction, a derivative of the attributable risk percent, is computed as (OR-1)/OR, and is interpreted as the proportion of variants in cases that are causative of the disease, or the probability that a randomly selected variant (in a case)

***Table 6a*** Prevalences and odds ratios are shown for the whole myosin protein, and for pre-specified regions of interest: the head (defined in (Walsh et al., 2016)), the mesa surface & converter "sphere" region (in the pre-stroke state; (Homburger et al., 2016)), the IHM-related interactions, and the motor domain functional residues. *"RoI"* = region of interest. p-value for all comparisons is < 1x10^-40^.

| region | n case variants in RoI | case prevalence | n control variants in RoI | control prevalence | odds ratio (OR) | OR 95% CI | etiological fraction |
| --- | --- | --- | --- | --- | --- | --- | --- |
| **All myosin** | 854 | 0.1400 | 449 | 1.35e-02 | 11.90 | 10.6-13.4 | 0.916 |
| head cluster | 641 | 0.1050 | 101 | 3.03e-03 | 38.60 | 31.2-48.2 | 0.974 |
| not head | 213 | 0.0348 | 348 | 1.04e-02 | 3.43 | 2.87-4.08 | 0.708 |
| **All IHM interactions** | 457 | 0.0748 | 85 | 2.55e-03 | 31.60 | 25-40.4 | 0.968 |
| priming | 171 | 0.0280 | 15 | 4.50e-04 | 64.00 | 37.7-117 | 0.984 |
| anchoring | 109 | 0.0178 | 38 | 1.14e-03 | 15.90 | 10.9-23.7 | 0.937 |
| stabilizing | 241 | 0.0394 | 26 | 7.79e-04 | 52.60 | 35-82.3 | 0.981 |
| scaffolding | 147 | 0.0241 | 16 | 4.80e-04 | 51.40 | 30.6-92.4 | 0.981 |
| **converter sphere (pre-stroke)** | 81 | 0.0133 | 3 | 8.99e-05 | 150.00 | 49.3-737 | 0.993 |
| **mesa surface (pre-stroke)** | 293 | 0.0479 | 40 | 1.20e-03 | 42.00 | 30-59.9 | 0.976 |
| **MD functional sites** | 164 | 0.0268 | 14 | 4.20e-04 | 65.70 | 38-123 | 0.985 |

***Table 6b*** Equivalent data is shown for all IHM interaction regions as defined in Supplementary file 2. Prevalence at interactions f1 (bh), a (fh) and h (bh) does not differ significantly. For other comparisons p < 1x10^-7^.

| IHM interaction | n case variants in RoI | case prevalence | n control variants in RoI | control prevalence | odds ratio (OR) | OR 95% CI | etiological fraction |
| --- | --- | --- | --- | --- | --- | --- | --- |
| g (bh) | 30 | 0.004910 | 0 | 0.00e+00 | Inf | 41.9-Inf | 1.000 |
| d1 (bh) | 32 | 0.005240 | 0 | 0.00e+00 | Inf | 44.9-Inf | 1.000 |
| i (bh) | 44 | 0.007200 | 2 | 5.99e-05 | 121.00 | 31.6-1020 | 0.992 |
| d2 (fh) | 101 | 0.016500 | 5 | 1.50e-04 | 112.00 | 46.4-354 | 0.991 |
| f1 (tail) | 57 | 0.009330 | 3 | 8.99e-05 | 105.00 | 34-522 | 0.990 |
| f2 (tail) | 57 | 0.009330 | 3 | 8.99e-05 | 105.00 | 34-522 | 0.990 |
| j (bh) | 56 | 0.009160 | 3 | 8.99e-05 | 103.00 | 33.4-513 | 0.990 |
| f2 (bh) | 50 | 0.008180 | 3 | 8.99e-05 | 91.70 | 29.6-459 | 0.989 |
| rlc-mhc (bh) | 12 | 0.001960 | 1 | 3.00e-05 | 65.60 | 9.7-2760 | 0.985 |
| elc-mhc (bh) | 136 | 0.022300 | 14 | 4.20e-04 | 54.20 | 31.2-102 | 0.982 |
| d1 (fh) | 40 | 0.006540 | 5 | 1.50e-04 | 44.00 | 17.3-143 | 0.977 |
| rlc-mhc (fh) | 13 | 0.002130 | 2 | 5.99e-05 | 35.60 | 8.04-324 | 0.972 |
| e (bh) | 30 | 0.004910 | 5 | 1.50e-04 | 32.90 | 12.6-109 | 0.970 |
| a (tail) | 29 | 0.004740 | 5 | 1.50e-04 | 31.80 | 12.2-105 | 0.969 |
| g (tail) | 33 | 0.005400 | 7 | 2.10e-04 | 25.90 | 11.2-69.2 | 0.961 |
| elc-mhc (fh) | 50 | 0.008180 | 12 | 3.60e-04 | 22.90 | 12-47.4 | 0.956 |
| d2 (bh) | 9 | 0.001470 | 4 | 1.20e-04 | 12.30 | 3.43-54.7 | 0.919 |
| f1 (bh) | 1 | 0.000164 | 2 | 5.99e-05 | 2.73 | 0.0463-52.4 | 0.634 |
| a (fh) | 1 | 0.000164 | 2 | 5.99e-05 | 2.73 | 0.0463-52.4 | 0.634 |
| h (bh) | 9 | 0.001470 | 33 | 9.89e-04 | 1.49 | 0.627-3.18 | 0.329 |

***Table 6c*** Present combined data from 7 interactions with individual EF > 0.99.

| n case variants in RoI | case prevalence | n control variants in RoI | control prevalence | odds ratio (OR) | OR 95% CI | etiological fraction |
| --- | --- | --- | --- | --- | --- | --- |
| 377 | 0.0617 | 16 | 0.00048 | 137 | 83.4-242 | 0.993 |

***Table 6d***. Equivalent data is shown for all motor domain functional sites. All comparisons are significant (p < 1x10^-15^.)

| md function | n case variants in RoI | case prevalence | n control variants in RoI | control prevalence | odds ratio (OR) | OR 95% CI | etiological fraction |
| --- | --- | --- | --- | --- | --- | --- | --- |
| converter | 100 | 0.01640 | 3 | 8.99e-05 | 184.0 | 61.5-921 | 0.995 |
| relay | 14 | 0.00229 | 1 | 3.00e-05 | 76.6 | 11.6-3180 | 0.987 |
| Actin-myosin | 37 | 0.00605 | 7 | 2.10e-04 | 29.0 | 12.8-77.2 | 0.966 |
| nucleotideBinding | 13 | 0.00213 | 3 | 8.99e-05 | 23.7 | 6.51-129 | 0.958 |

***Table 6e.***  Variants in ExAC are not over-represented at IHM sites or MD functional sites. They are significantly depleted at MD sites, and are nominally (though not significantly) depleted for each IHM interaction type.

| interaction | variants in interaction site | rate | length of interaction site (aa) | expected rate | rate ratio | p_binom_ |
| --- | --- | --- | --- | --- | --- | --- |
| **all IHM interactions** | 50 | 0.1980 | 447 | 0.2310 | 0.857 | 0.23300 |
| priming | 12 | 0.0474 | 113 | 0.0584 | 0.812 | 0.59000 |
| anchoring | 18 | 0.0711 | 156 | 0.0806 | 0.882 | 0.64500 |
| stabilizing | 18 | 0.0711 | 189 | 0.0977 | 0.728 | 0.16900 |
| scaffolding | 9 | 0.0356 | 120 | 0.0620 | 0.574 | 0.08910 |
| **MD functional residues** | 11 | 0.0435 | 194 | 0.1000 | 0.435 | 0.00154 |
