# Supplementary material for: Effects of myosin variants on interacting-heads motif explain distinct hypertrophic and dilated cardiomyopathy phenotypes
Source: eLife. 2017 Jun 13;6:e24634. doi: 10.7554/eLife.24634 (PMC5469618; doi:10.7554/eLife.24634)
Supplement: Supplementary file 7. — DOI: http://dx.doi.org/10.7554/eLife.24634.035 [file elife-24634-supp7.docx]

**Supplementary file 7. Leveraging regional distribution for the clinical interpretation of DCM-causing variants.** The prevalence of rare (ExAC global AF < 1x10^-4^) missense variants in *MYH7* in 1315 DCM cases and ExAC controls (median number genotype per site = 33364) are compared using the binomial test. The etiological fraction, a derivative of the attributable risk percent, is computed as (OR-1)/OR, and is interpreted as the proportion of variants in cases that are causative of the disease, or the probability that a randomly selected variant (in a case).

Prevalences and odds ratios are shown for the whole myosin protein, and for pre-specified regions of interest: the head (defined in (Walsh et al., 2016)), the mesa surface & converter "sphere" region (in the pre-stroke state; (Homburger et al., 2016)), the IHM-related interactions, and the motor domain functional residues. *"RoI"* = region of interest.

Variants at sites of IHM-priming interactions, and at MD functional sites (driven by a signal at the nucleotide binding pocket), have a probability of pathogenicity > 0.95, which is nominally sufficient for an interpretation of "likely Pathogenic", though in practice many laboratories would require a higher confidence for a clinically actionable assertion of pathogenicity.

| region | n case variants in RoI | case prevalence | n control variants in RoI | control prevalence | pBinom | odds ratio (OR) | OR 95% CI | etiological fraction |
| --- | --- | --- | --- | --- | --- | --- | --- | --- |
| **All myosin** | 68 | 0.05170 | 449 | 1.35e-02 | 3.03e-20 | 4.000 | 3.03-5.2 | 0.750 |
| head cluster | 36 | 0.02740 | 101 | 3.03e-03 | 1.52e-22 | 9.270 | 6.13-13.7 | 0.892 |
| not head | 32 | 0.02430 | 348 | 1.04e-02 | 1.64e-05 | 2.370 | 1.59-3.42 | 0.578 |
| **All IHM interactions** | 21 | 0.01600 | 85 | 2.55e-03 | 7.67e-11 | 6.350 | 3.73-10.4 | 0.843 |
| priming | 13 | 0.00989 | 15 | 4.50e-04 | 9.48e-14 | 22.200 | 9.7-50.2 | 0.955 |
| anchoring | 1 | 0.00076 | 38 | 1.14e-03 | 1.00e+00 | 0.667 | 0.0165-3.96 | -0.499 |
| stabilizing | 4 | 0.00304 | 26 | 7.79e-04 | 2.05e-02 | 3.910 | 0.991-11.3 | 0.744 |
| scaffolding | 5 | 0.00380 | 16 | 4.80e-04 | 4.91e-04 | 7.960 | 2.28-22.8 | 0.874 |
| **converter sphere (pre-stroke)** | 0 | 0.00000 | 3 | 8.99e-05 | 1.00e+00 | 0.000 | 0-61.3 | -Inf |
| **mesa surface (pre-stroke)** | 6 | 0.00456 | 40 | 1.20e-03 | 5.60e-03 | 3.820 | 1.32-9.09 | 0.738 |
| **MD functional sites** | 13 | 0.00989 | 14 | 4.20e-04 | 4.01e-14 | 23.800 | 10.3-54.7 | 0.958 |
| nucleotide binding | 8 | 0.00608 | 3 | 8.99e-05 | 8.36e-13 | 68.000 | 16.3-396 | 0.985 |
